# Targeting the hexosamine biosynthetic pathway and O-linked N-acetylglucosamine cycling for therapeutic and imaging capabilities in diffuse large B-cell lymphoma

**DOI:** 10.18632/oncotarget.12413

**Published:** 2016-10-03

**Authors:** Lan V. Pham, Jerry L. Bryant, Richard Mendez, Juan Chen, Archito T. Tamayo, Zijun Y. Xu, Ken H. Young, Ganiraju C. Manyam, David Yang, L. Jeffrey Medeiros, Richard J. Ford

**Affiliations:** ^1^ Department of Hematopathology, The University of Texas MD Anderson Cancer Center, Houston, TX, USA; ^2^ Division of Translational Medicine, Cell>Point Pharmaceuticals, Centennial, CO, USA; ^3^ Department of Bioinformatics and Computational Biology, The University of Texas MD Anderson Cancer Center, Houston, TX, USA

**Keywords:** DLBCL, hexosamine, NF-κB, NFAT, O-linked N-acetylglucosamine

## Abstract

The hexosamine biosynthetic pathway (HBP) requires two key nutrients glucose and glutamine for O-linked N-acetylglucosamine (O-GlcNAc) cycling, a post-translational protein modification that adds GlcNAc to nuclear and cytoplasmic proteins. Increased GlcNAc has been linked to regulatory factors involved in cancer cell growth and survival. However, the biological significance of GlcNAc in diffuse large B-cell lymphoma (DLBCL) is not well defined. This study is the first to show that both the substrate and the endpoint O-GlcNAc transferase (OGT) enzyme of the HBP were highly expressed in DLBCL cell lines and in patient tumors compared with normal B-lymphocytes. Notably, high *OGT* mRNA levels were associated with poor survival of DLBCL patients. Targeting OGT via small interference RNA in DLBCL cells inhibited activation of GlcNAc, nuclear factor kappa B (NF-κB), and nuclear factor of activated T-cells 1 (NFATc1), as well as cell growth. Depleting both glucose and glutamine in DLBCL cells or treating them with an HBP inhibitor (azaserine) diminished O-GlcNAc protein substrate, inhibited constitutive NF-κB and NFATc1 activation, and induced G0/G1 cell-cycle arrest and apoptosis. Replenishing glucose-and glutamine-deprived DLBCL cells with a synthetic glucose analog (ethylenedicysteine-N-acetylglucosamine [ECG]) reversed these phenotypes. Finally, we showed in both *in vitro* and *in vivo* murine models that DLBCL cells easily take up radiolabeled technetium-99m-ECG conjugate. These findings suggest that targeting the HBP has therapeutic relevance for DLBCL and underscores the imaging potential of the glucosamine analog ECG in DLBCL.

## INTRODUCTION

Diffuse large B-cell lymphoma (DLBCL) is the most frequent non-Hodgkin lymphoma histotype clinically, with approximately 30,000 new cases/year in the United States. Although DLBCL is initially responsive to standard frontline rituximab, cyclophosphamide, doxorubicin, vincristine, prednisone (R-CHOP) chemoimmunotherapy (~80% partial or complete response), the disease frequently relapses; almost half of all patients with DLBCL are not cured by either chemotherapy or stem cell transplantation and experience relapse or display primary refractory disease with shortened survival [1]. Therefore, new novel therapeutic approaches are urgently needed for patients with relapsed/refractory (R/R) DLBCL.

Because cancer cells preferentially utilize aerobic glycolysis as the major source of energy for growth and survival, this pathway has become a relevant potential therapeutic target in various cancers, including aggressive B-cell lymphomas [2]. Aerobic glycolysis (i.e., the Warburg effect) is characterized by increased glycolysis and lactate production despite sufficient oxygen availability. Aerobic glycolysis in cancer cells is often defined by excessive cellular glucose uptake, which is readily quantifiable *in vitro* and *in vivo* [3, 4].

Glucose metabolism provides a major source of energy for tumor cell growth and survival and is the basis for clinical 18F-fluorodeoxyglucose–PET imaging in various cancers, including DLBCL [3-5]. Various studies have shown that 18F-fluorodeoxyglucose–PET/computed tomography imaging has prognostic value and can assess DLBCL progression and survival after rituximab immunotherapy [6, 7], suggesting that glucose metabolism plays a key role in the pathogenesis of the disease process. However, the extent to which glucose metabolism contributes to the maintenance and progression of DLBCL remains unclear.

Cancer cells also consume large amounts of glutamine, a key amino acid involved in protein synthesis–dependent tumor cell growth [8, 9]. Among its various roles, glutamine is a precursor amino acid for the synthesis of glucosamine, a prominent initiator in the hexosamine biosynthetic pathway (HBP) [10]. Fructose-6-phosphate from the glycolytic pathway combines with glutamine in the presence of the enzyme glutamine–fructose-6-phosphate amidotransferase (GFAT) to synthesize glucosamine-6-phosphate. Subsequent enzymatic reactions lead to the production of uridine diphosphate N-acetylglucosamine (GlcNAc), a substrate for O-linked glycosylation regulated by the endpoint enzyme O-linked GlcNAc (O-GlcNAc) transferase (OGT). OGT is the enzyme that catalyzes the addition of a single GlcNAc residue to the hydroxyl groups of serine and/or threonine residues of target proteins. The HBP, which ends in O-GlcNAc cycling (O-GlcNAcylation), has been implicated in cellular signaling and regulation of transcription factors involved in cancer biology [11-14]. The biological significance of the HBP in the pathogenesis of DLBCL is not known. However, recent studies have indicated that these pathways might be linked to glycolysis that could be involved in the pathogenesis of several types of cancers [15-18]. Determining how altered O-GlcNAc cycling and glucose/glutamine metabolisms contribute to refractory DLBCL phenotypes could provide specific therapeutic strategies for this disease.

In this study, we hypothesized that the HBP and O-GlcNAc metabolism play critical roles in the regulation of DLBCL cell proliferation and survival, and that this mechanism might be a candidate for therapeutic targeting. We found that the increased glucose and glutamine consumption by DLBCL cells feeds into the HBP, which in turn enhances nuclear retention of the transcription factors nuclear factor kappa B (NF-κB) and nuclear factor of activated T-cells 1 (NFATc1) through GlcNAc changes. We demonstrated that OGT was highly expressed in both DLBCL cell lines and primary tumor cells from patients. We observed that high *OGT* mRNA expression was associated with poor survival of DLBCL patients. We also demonstrated that depleting both glucose and glutamine in DLBCL cells or treating cells with an HBP inhibitor (azaserine) diminished O-GlcNAc protein substrate levels, inhibited constitutive NF-κB and NFATc1 activation, and induced G0/G1 cell-cycle arrest and apoptosis. Replenishing glucose- and glutamine-deprived DLBCL cells with a synthetic glucose analog (ethylenedicysteine-N-acetylglucosamine [ECG]) reversed these phenotypes. Finally, we showed in both *in vitro* and *in vivo* models that DLBCL cells can easily take up radiolabeled technetium-99m-ECG (^99m^Tc-ECG) conjugate. Our findings suggest that targeting the HBP is a novel therapeutic strategy that can exploit the persistent glucose/glutamine addiction of DLBCL cells.

## RESULTS

### OGT expression is increased in DLBCL cells, and high *OGT* mRNA expression is associated with poor prognosis in DLBCL patients

**Figure 1:**
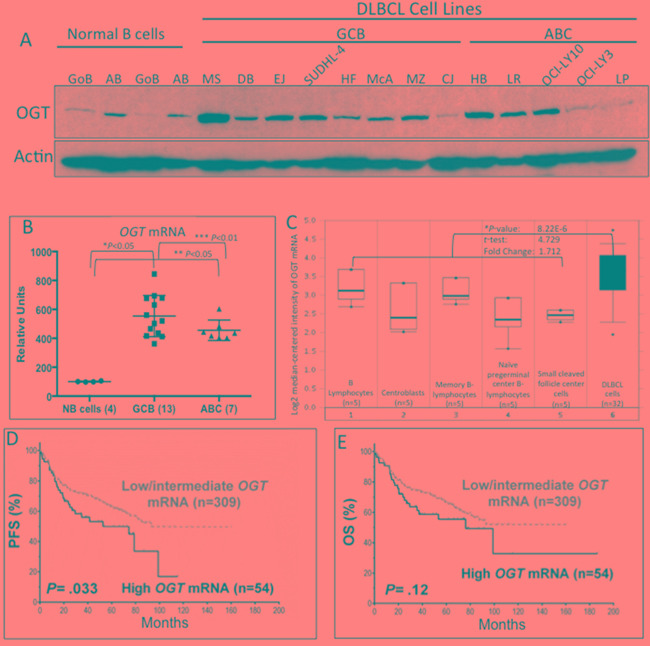
Increased OGT expression in diffuse large B-cell lymphoma (DLBCL cells) and association with poor patient survival **A.** Western blot results demonstrate that most DLBCL cell lines have higher protein expression of O-linked N-acetylglucosamine transferase (OGT) than normal B-lymphocytes. Abbreviations: GoB, unstimulated B-lymphocytes; AB, activated B-lymphocytes; SU4, SUDHL-4; LY3, OCI-LY3; LY10, OCI-LY10; GCB, germinal center–derived B-lymphocytes; ABC, activated B-cell. Normal B-cells were obtained from blood samples from two different healthy donors. **B.** Gene expression profiling of *OGT* mRNA in DLBCL cell lines and normal B-cells. Abbreviations: NB, normal B-cells. *Comparison between normal B-cells and GCB DLBCL cell lines; **comparison between normal B-cells and ABC DLBCL cell lines; ***comparison between GCB and ABC DLBCL cell lines. **C.** mRNA expression in primary DLBCL and NB using the Oncomine database. The Student *t*-test was applied to the Oncomine results. The boxes represent the 25th through 75th percentiles. The horizontal lines represent the medians. The whiskers represent the 10th and 90th percentiles, and the asterisks represent the ranges. 1, B-lymphocytes (n=5); 2, centroblasts (n=5); 3, memory B-lymphocytes (n=5); 4, naïve pregerminal center B-lymphocytes (n=5); 5, small cleaved follicle center cells (n=5); 6, DLBCL cells (dark shade; n=32). *Comparison between the average of all B cell stages vs. DLBCL primary cells. **D and E.** Patients with de novo DLBCL and high *OGT* mRNA levels showed a trend for poor progression-free survival (PFS) and overall survival (OS)

To assess the importance of the HBP in cellular growth and survival of DLBCL cells, we analyzed OGT protein and mRNA expression in DLBCL cell lines, primary DLBCL tumor cells, and normal human B-lymphocytes. Figure 1A shows that in contrast to normal unstimulated and activated B-cells, most patient-derived germinal center-derived (GCB)–DLBCL and activated B-cell (ABC)–DLBCL cell lines expressed high levels of OGT protein. Similarly, we found that the *OGT* mRNA levels in DLBCL cell lines were significantly higher than in normal B-cells (*P*<0.5; Figure 1B).

We then analyzed the *OGT* mRNA expression in primary DLBCL cells using the publicly available Oncomine microarray database. Consistent with our data, a representative dataset [19] revealed that *OGT* mRNA levels were higher in primary DLBCL cells than in normal B-cells at different stages of development (Figure 1C). Moreover, when comparing the *OGT* gene expression profile of DLBCL with that of other types of cancers, we consistently noted that DLBCL is one of the few malignancies that exhibit high *OGT* mRNA expression levels (Supplementary Figure S1).

Next, we examined the clinical significance of *OGT* mRNA expression in a large cohort of de novo DLBCL patients treated with the standard R-CHOP regimen (n=363). To measure mRNA expression of the *OGT* gene from the GEP dataset, we retrieved the intensities of six OGT probe-sets and used the average value as the *OGT* mRNA expression levels. Patients with high OGT expression had the worst PFS (*P*=0.033; Figure 1D) and a trend suggestive of poor OS compared with patients with low/intermediate *OGT* mRNA expression (*P*=0.12; Figure 1E).

### OGT and O-GlcNAcylation control cell growth and survival mechanisms in DLBCL

**Figure 2:**
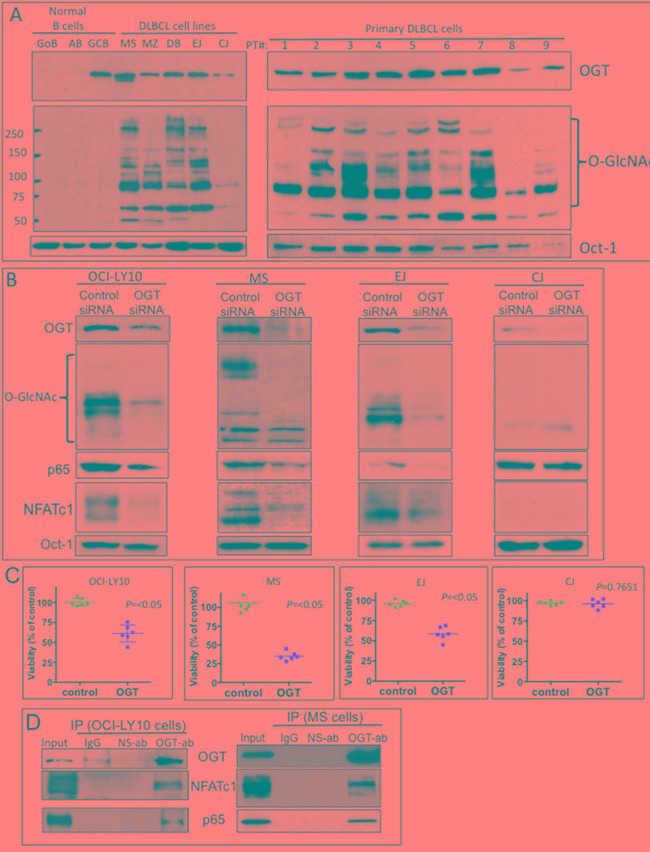
Elevated O-linked N-acetylglucosamine (O-GlcNAc) cycling (O-GlcNAcylation) and its association with nuclear factor of activated T-cells 1 (NFATc1) and nuclear factor kappa B (NF-κB)-p65 transcription factors in diffuse large B-cell lymphoma (DLBCL) **A.** Purified nuclear extracts from normal human B-lymphocytes, representative DLBCL cell lines, and primary DLBCL cells from patients (1–9) were subjected to Western blot analysis to determine expression of O-GlcNAc transferase (OGT), O-GlcNAc, and Oct-1 protein. Abbreviations: GoB, unstimulated B-lymphocytes; AB, activated B-lymphocytes; GCB, germinal center–derived B-lymphocytes. **B.** Four representative DLBCL cell lines were transfected with control small interfering RNA (siRNA) or a validated O-GlcNAc transferase (OGT) siRNA. After 48 h, nuclear extracts were purified and subjected to Western blot analysis for expression of OGT, O-GlcNAc, p65, NFATc1, and Oct-1 (loading control) protein. **C.** Transfected cells were also subjected to cell proliferation analysis using viability assays. Data is representative of two independent experiments with triplicate transfections (n=6). **D.** Immunoprecipitation assays using anti-OGT antibody show direct association of OGT and transcription factors NFATc1 and p65 in two representative DLBCL cell lines (OCI-LY10 and MS). Abbreviations: ab, antibody; IP, immunoprecipitation; IgG, immunoglobulin G; NS-ab, nonspecific antibody

Because O-GlcNAcylation is primarily regulated by OGT through the addition of GlcNAc on the serine or threonine residues of nucleocytoplasmic proteins, particularly key transcription factors involved in growth and survival mechanisms, we examined the nuclear acylation level of O-GlcNAc in DLBCL cells. Nuclear extracts were purified from normal B cell controls, five representative DLBCL cell lines, and nine primary DLBCL cases, and subjected to immunoblotting for OGT and O-GlcNAc protein expression. Both representative DLBCL cell lines and primary DLBCL tumor cells displayed higher levels of nuclear O-GlcNAcylation than those observed innormal B-cells (Figure 2A). Since the CJ cell line displayed a low level of OGT and O-GlcNAcylation in comparison to other DLBCL cell lines, we used this cell line as a control for subsequent experiments. These results suggest that the increased OGT and nuclear O-GlcNAcylation in DLBCL may play an important role in the biology and/or pathophysiology of DLBCL.

To determine the biological significance of the HBP and O-GlcNAcylation for the growth and survival of DLBCL, OGT expression in three representative DLBCL cell lines with high levels of OGT and GlcNAC (OCI-LY10, MS, EJ) and one DLBCL cell line with low levels of OGT and GlcNAC (CJ) was knocked down using a validated small interference RNA (siRNA) approach (Supplementary Figure S2). We found that OCI-LY10, MS and EJ OGT siRNA-transfected cells exhibited lower OGT protein expression than control siRNA-transfected cells, which correlated with decreased GlcNAc protein acylation levels (Figure 2B). The levels of both OGT and GlcNAC were initially low in CJ cells and were not significantly affected by OGT siRNA (Figure 2B).

We then investigated whether GlcNAc modifications have any effect on key transcription factors that regulate cell growth and survival of DLBCL. The transcription factors NF-κB-p65 and NFATc1 were previously shown to play key roles in the pathophysiology of DLBCL [20-22]. Additionally, a key study demonstrated that OGT is a central factor for T-and B-lymphocyte activation, which involves the modification of both NF-κB-p65 and NFATc1 through O-GlcNAc [23]. We discovered that OGT knockdown inhibited the nuclear expression of both NF-κB-p65 and NFATc1 proteins in OCI-LY10, MS and EJ but not in CJ cells (Figure 2B). The CJ cell line has high level of NF-κB-p65 that was not affected by OGT siRNA (Figure 2B), suggesting that a different mechanism for NF-κB regulation in this cell line. We also found that OGT siRNA transfected cells exhibited lower cell viability in OCI-LY10, MS and EJ but not in CJ cells (Figure 2C).

Next, we investigated whether the NF-κB-p65 and NFATc1 transcription factors interact with OGT. Co-immunoprecipitation assay results (Figure 2D) supported the potential interaction of NF-κB-p65 and NFATc1 with OGT in two representative DLBCL cell lines (OCI-LY10 and MS). These findings suggest that one of the mechanisms that controls activation of the transcription factors NF-κB-p65 and NFATc1 in DLBCL cells, could be through GlcNAc protein modifications.

### O-GlcNAcylation in DLBCL cells is glucose-and glutamine-dependent

**Figure 3:**
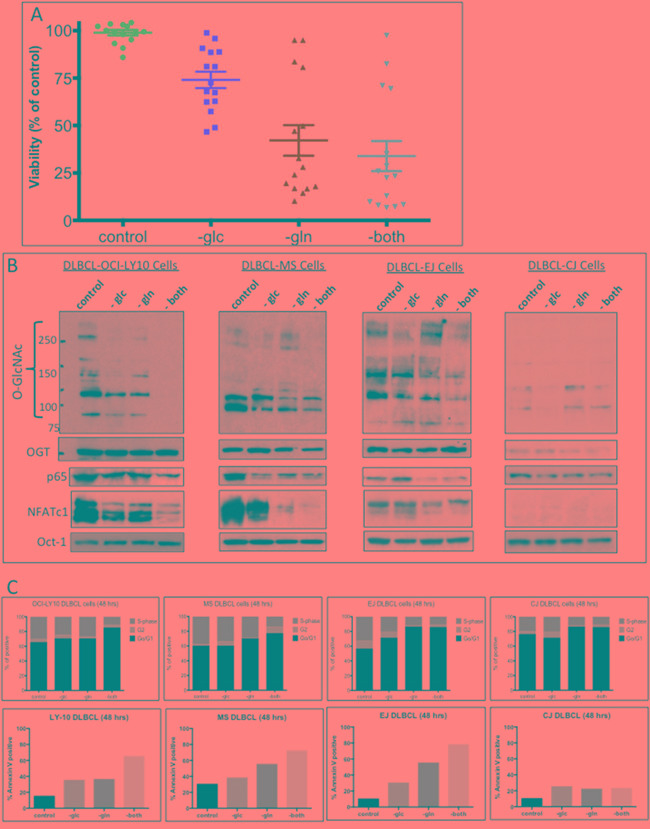
Increased uptake of glucose and glutamine by diffuse large B-cell lymphoma (DLBCL) cells correlates with N-acetylglucosamine (GlcNAc) protein modifications **A.** Fifteen representative DLBCL cell lines were cultured without glucose (-glc), glutamine (-gln), or both (-both); after 48 h, the cells were analyzed for viability. **B.** Representative DLBCL cells (OCI-LY10, MS, EJ, and CJ) were cultured with or without glucose (-glc), glutamine (-gln), or both (-both) for 48 h. Nuclear extracts were purified and were subjected to Western blot analysis to determine O-linked GlcNAc (O-GlcNAc) protein modification and expression of p65, nuclear factor of activated T-cells 1 (NFATc1), and O-GlcNAc transferase (OGT) protein. **C.** Representative DLBCL cell lines (OCI-LY10, MS, EJ, and CJ) were incubated for 48 h without glucose (-glc), glutamine (-gln), or both (-both). Cell-cycle analysis (top panels) and apoptosis assays (bottom panels) were performed

Glucose and glutamine are key nutrients for cancer cells and are precursor substrates in the synthesis of GlcNAc via the HBP. To determine the potential role of glucose and glutamine in the HBP and GlcNAcylation in DLBCL cells, we investigated whether depriving DLBCL cells of glucose, glutamine, or both would affect GlcNAc, NF-κB-p65, NFATc1 levels, and influence cell growth/survival outcomes. Figure 3A shows that although the majority of the 15 representative DLBCL cell lines were more dependent on glutamine than glucose, cell viability was more significantly decreased when the cells were deprived of both nutrients. Cell viability in several cell lines, including the CJ cell line, was not affected after nutrients deprivation. Concomitantly, depriving representative DLBCL cell lines with high OGT/GlcNAC activities (OCI-LY10, MS, and EJ) of both glucose and glutamine diminished nuclear GlcNAc protein acyl modifications (Figure 3B). Furthermore, removal of both glucose and glutamine also led to the downregulation of both nuclear NF-κB-p65 and NFATc1 in DLBCL cells (Figure 3B). These activities were not seen after nutrient deprivation in CJ cells (Figure 3B).

Next, we investigated whether glucose and glutamine deprivation affected cell-cycle progression and survival, using representative cell lines with high (EJ, MS, and OCI-LY10) or low (CJ) GlcNAc protein modification levels. Figure 3C shows that removal of both glucose and glutamine from cell lines EJ, MS, and OCI-LY10 resulted in increased G0/G1 cell-cycle arrest and apoptosis. In contrast, removal of both glucose and glutamine from the cell line CJ, had very little effect on the cell cycle or apoptosis (Figure 3C, right panels). Our results suggest that glucose- and glutamine-dependent GlcNAc protein modifications may play an important role in cell growth and survival in most DLBCL.

### Pharmacologic targeting of the HBP in DLBCL inhibits cell growth and survival

**Figure 4:**
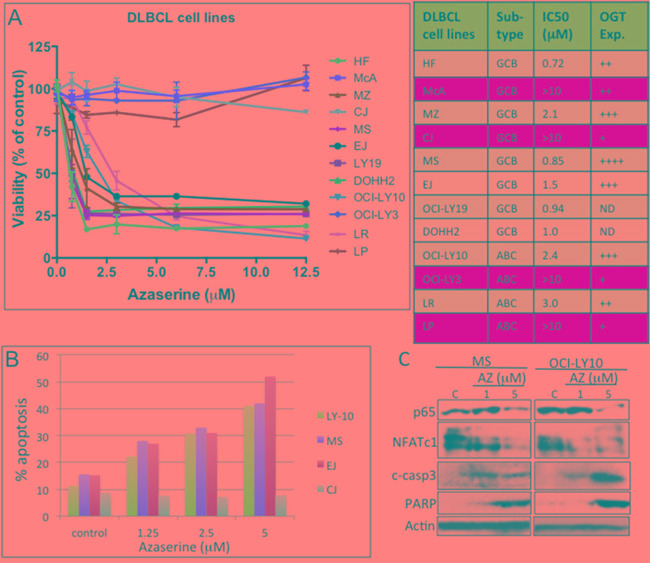
Pharmacologic targeting of the hexosamine biosynthetic pathway (HBP) in diffuse large B-cell lymphoma (DLBCL) **A.** Representative DLBCL cell lines were inhibited with various concentrations of the glutamine–fructose-6-phosphate amidotransferase (GFAT) inhibitor azaserine. Cell viability assays were performed 72 h after inhibition. Abbreviations: IC_50_, median inhibitory concentration. OGT protein expression level: +, low; ++, intermediate; +++, high; ++++, very high. ND, no data; Exp., expression. **B.** Representative DLBCL cell lines OCI-LY10, MS, EJ, and CJ were treated with Azaserine in a dose-dependent manner, and Annexin V staining/FACS analysis was assessed to determine cell undergoing apoptosis after 48 h of treatment. **C.** Representative DLBCL cell lines (MS and OCI-LY10) were treated with increasing concentration of Azaserine (AZ; 1 and 5 μM) for 48 h. Protein extracts were purified and used in Western blotting for p65, NFATc1, cleaved caspase 3, PARP, and actin (loading control)

We then investigated whether inhibiting the HBP pathway would alter DLBCL cell growth and survival and thus represent a potential target for DLBCL treatment. To target the HBP pharmacologically, we utilized azaserine, a competitive inhibitor of GFAT. We conducted viability assays on 12 representative DLBCL cell lines with increasing concentrations of azaserine. Cell lines with high OGT protein expression were more sensitive to azaserine, whereas cell lines with low OGT expression level (McA, CJ, LY-3, and LP) were less sensitive to azaserine (Figure 4A).

We next determined the effects of azaserine on apoptosis in four representative DLBCL cell lines (EJ, MS, OCI-LY10, and CJ). Azaserine treatment induces apoptosis in EJ, MS, and OCI-LY10 DLBCL cells but not in CJ cells (Figure 4B). Azaserine treatment also inhibited both p65 and NFATc1 protein expression, subsequently leading to the activation apoptotic related proteins, cleaved caspase 3 and PARP (Figure 4C). These results suggest that azaserine can block NF-κB-p65 and NFATc1 in DLBCL cells, which subsequently leads the induction of apoptosis, similar to what was observed in the OGT knockdown and nutrient depletion approaches.

### ^99m^Tc-ECG imaging distinguishes DLBCL from muscle tissue *in vivo*

**Figure 5:**
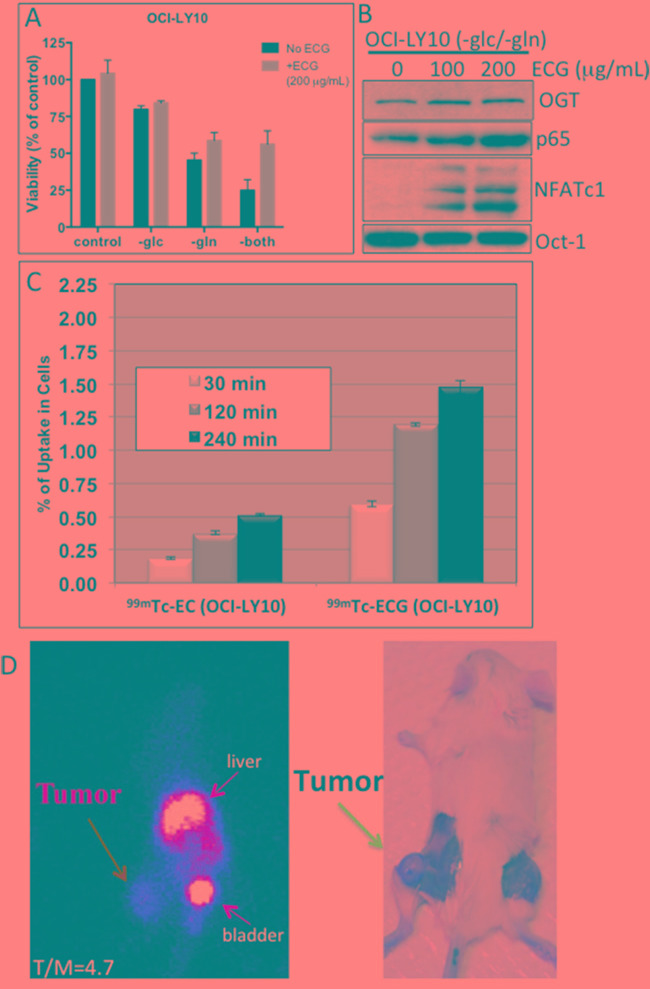
*In vitro* technetium-99m-ethylenedicysteine-N-acetylglucosamine (99mTc-ECG) uptake in diffuse large B-cell lymphoma (DLBCL) cells and *in vivo* 99mTc-ECG imaging in a severe combined immunodeficiency mouse lymphoma model **A.** OCI-LY10 cells were cultured without glucose (-glc), glutamine (-gln) or both (-both) and supplemented with ECG (+ECG; 200 μg/ml) or without ECG (No ECG). Viability was assessed after 48 h of incubation. **B.** OCI-LY10 cells were cultured without glucose and glutamine and were supplemented with various doses of ECG. After 48 h, purified nuclear extracts were subjected to Western blot analysis for O-linked N-acetylglucosamine transferase (OGT), nuclear factor kappa B (NF-κB)-p65, nuclear factor of activated T-cells 1 (NFATc1), and Oct-1 (loading control). **C.** *In vitro* uptake of ^99m^Tc-ECGin a representative DLBCL cell line (OCI-LY10). **D.** Planar scintigraphy ^99m^Tc-ECG image (left panel) shows a hightumor-to-muscle (T/M) ratio at 120 min (left panel), and a necropsy image of the same mouse shows the tumor on the left thigh (right panel)

GlcNAc residue, like glucose and glutamine, can be taken up by cells through transporters and thus is an attractive target for imaging. The chelator ethylenedicysteine (EC) was previously conjugated to glucosamine to develop a GlcNAc analog called EC-glucosamine (ECG) [24]. Radiolabeled ^99m^Tc-ECG has shown promising imaging capabilities in various rodent experimental tumor models [24, 25]. To determine whether ECG can be utilized as a potential imaging agent for DLBCL, we first examined the uptake of ECG in DLBCL cells. Our initial experiments showed that replenishing ECG in glucose- and glutamine-deprived OCI-LY10 DLBCL cells stimulated cell viability (Figure 5A) and induced both NF-κB-p65 and NFATc1 activation (Figure 5B), suggesting that ECG can incorporate into the HBP through GlcNAc protein modification. *In vitro* uptake experiments indicated that DLBCL OCI-LY10 cells (Figure 5C) and two other representative DLBCL cell lines (Supplementary Figure S3A) had greater uptake of radiolabeled ^99m^Tc-ECG than control ^99m^Tc-EC.

To investigate whether ^99m^Tc-ECG can differentiate tumors from muscle tissue, we used a OCI-LY10 DLBCL xeno-transplant SCID mouse model. OCI-LY10 cells were injected subcutaneously into the thigh of each mouse's left hind leg. Planar scintigraphic imaging of ^99m^Tc-ECG was performed 14–20 days after inoculation when tumors reached approximately 1 cm in diameter (Figure 5D, right panel). A selected planar scintigraphic image of a representative mouse shows the uptake of ^99m^Tc-ECG in the tumor mass, with a tumor/muscle ratio of 4.7 after 120 min (Figure 5D, left panel). The high intensity isotope uptake level in the liver and bladder indicates the clearance of the labeled ECG. The average tumor/muscle ratio for four mice was 3.14 (Supplementary Figure S3B).

## DISCUSSION

We found that DLBCL cells use both glucose and glutamine, which feed into the HBP and trigger O-GlcNAcylation and activate NF-κB-p65 and NFATc1, to regulate DLBCL cell growth and survival. Previous studies have demonstrated the chronic activation of the HBP and GlcNAc in various cancers [26, 27], and our results show that increased glucose and glutamine uptake by DLBCL cells keeps the HBP and O-GlcNAc in a hyperactive state. We predict that chronically active HBP and GlcNAc modifications promote and maintain a malignant phenotype, and our findings could help identify novel DLBCL diagnostic biomarkers as well as therapeutic targets. These include the enzyme OGT, whose high expression in many DLBCL cells but apparently not in normal B-lymphocytes predicts a poor clinical outcome. These findings suggest that the development of pharmacological agents that target the HBP, particularly the OGT enzyme, could have a significant therapeutic effect on DLBCL. There are no OGT inhibitors currently being tested in the clinic for cancer patients, indicating that future research is needed to develop effective OGT inhibitors.

Over the last decade, focus on cancer metabolism research has enhanced our understanding of aerobic glycolysis, which involves high avidity for glucose and glutamine. Recent studies have shown that cancer progression and persistence are accompanied by increased glutamine metabolism, providing the carbon and nitrogen sources required in anabolic pathways [28, 29]. The key metabolites from glutamine metabolism are important substrates of the HBP, which are converted to uridine diphosphate-GlcNAc, a carbohydrate post-translational modification of various nuclear and cytosolic proteins. Many oncogenic and tumor suppressor genes involved in key signaling pathways that control growth and survival mechanisms are, in fact, regulated through the HBP in the reprogramming of cellular metabolism [30]. In the current study, we have demonstrated that in DLBCL, GlcNAc mobilizes transcription factors via glucose and glutaminemetabolism and that inhibiting O-GlcNAc activity by removing both glucose and glutamine inhibits constitutive NFATc1 and NF-κB activation. Mechanisms of NF-κB-p65 and NFATc1 nuclear translocations also involve phosphorylation and dephosphorylation, respectively, but it is still unclear whether GlcNAC protein modification precedes phosphorylation and dephosphorylation or vice versa. NF-κB and NFATc1 transcription factors are known downstream targets of both the HBP and the B cell receptor (BCR) pathways [23, 31]. In DLBCL, these pathways are known to be constitutively activated in ABC histologic subtypes [32, 33]. However, our previous studies and several other studies have indicated that these transcription factors are also shown to be activated in some GCB-DLBCL cellular histotypes [21, 34, 35]. Future studies are needed to determine the interactions between post-tranlstional protein modifications through GlcNAC, phosphorylation, and dephosphorylation of NFATc1 and NF-κB transcription factors.

The imaging agent ^99m^Tc-ECG, also known as ^99m^Tc-Oncardia, is already used in clinical trials for various cancers and has shown great potential to become the next-generation theranostic imaging technology. The therapeutic and diagnostic capabilities of ^99m^Tc-ECG imaging for refractory DLBCL and other types of metabolically active cancers are promising. Such an approach has great potential to improve diagnosing and treating cancer and enhancing the quality and extending the life of patients with many types of cancer.

To our knowledge, this study is the first to link OGT and O-GlcNAc to DLBCL cell growth and survival mechanisms, which in turn affects NFATc1 and NF-κB-p65 transcription functions. Thus, the nutrient-sensing dynamics of OGT and the HBP may negatively affect normal metabolic states, which ultimately leads to cancer. Pharmacological intervention using enzymes that regulate O-GlcNAcylation may be a novel therapeutic strategy for patients with DLBCL. Finally, *in vitro* and *in vivo* uptake of the radio-labeled ECG (^99m^Tc-ECG) in DLBCL cells underscores the imaging potential of the HBP pathway in many types of cancer cells.

## MATERIALS AND METHODS

### Cell lines

Human DLBCL cell lines (MS, DS, DB, JM [McA], FN, EJ, HF, HB, MZ, LR, PL, CJ, LP) have been previously described and characterized [34]. The U-2932, OCI-LY19, DOHH2, Pfeiffer, SUDHL-4, OCI-LY10, and OCI-LY3 DLBCL cell lines were obtained from outside sources. Before conducting these experiments, we tested all cell lines for *Mycoplasma* using a MycoTect kit (Thermo Fisher Scientific, Waltham, MA) and validated the cell lines using short tandem repeats DNA fingerprinting at the Characterized Cell Line Core Facility at The University of Texas MD Anderson Cancer Center (Houston, TX). Stocks of authenticated cell lines were stored in liquid nitrogen for future use, and all cell lines used in this manuscript were from these authenticated stocks. Primary DLBCL cells were obtained using a protocol approved by the institution review board at MD Anderson Cancer Center. This study was conducted in accordance with the Helsinki protocol and approved by the Institutional Review Board of The University of Texas M. D. Anderson Cancer Center. The cells were cultured in Roswell Park Memorial Institute-1640 medium (Thermo Fisher Scientific) containing 15% fetal calf serum (HyClone Laboratories, Logan, UT) and 1% penicillin/streptomycin. Normal human B-lymphocytes were purified from healthy donor buffy coats, using the human B-cell enrichment cocktail from STEMCELL Technologies (Vancouver, BC, Canada). Purified B-cells were activated by incubation for 48 h with recombinant human CD40L and anti-immunoglobulin M (3.5 μg/mL; ICN Biomedicals, Santa Ana, CA). Germinal center–derived B-lymphocytes were purified from reactive tonsils.

### Gene expression profiling and DLBCL patients

Our study included 460 patients with de novo DLBCL treated with standard R-CHOP immunochemotherapy as previously described [36]. The diagnosis, review process, and cell-of-origin classification were carried out using gene expression profiling (GEP) or the immunohistochemical algorithms of Visco-Young and/or Choi, which have been previously described [37, 38]. Total RNA a was extracted from formalin-fixed paraffin-embedded tissues and was subjected to GEP using an Affymetrix GeneChips array (Santa Clara, CA). A total of 109 cases were excluded owing to poor GEP data.

Survival analysis was stratified by the *OTG* mRNA expression levels (Log2 values retrieved from the GEP data). The mean values of six probe-sets (209240_at, 212307_s_at, 220594_at, 229787_s_at, 207563_s_at, and 207564_x_at) for each patient were used for the *OGT* mRNA levels. Patients initially were divided into three groups for survival analysis according to the mean values of *OGT* mRNA expression: low mRNA (< mean – 1 standard deviation), high mRNA (> mean + 1 standard deviation), and intermediate mRNA (the remaining cases). Because there was no significant difference between the low and intermediate *OGT* mRNA groups, we combined these groups.

### Antibodies and reagents

OGT, GlcNAc, NFATc1, cleaved-caspase 3, PARP, and Oct-1 antibodies were purchased from Santa Cruz Biotechnology (Dallas, TX). NF-κB-p65 antibody was purchased from EMD Millipore (Billerica, MA). Azaserine was purchased from Sigma-Aldrich (St. Louis, MO). ECG was provided by Cell>Point (Centennial, CO). Silencer select pre-designed and validated OGT siRNA was obtained from Thermo Fisher Scientific.

### Immunoblot analysis

Lymphoma cells were washed twice in ice-cold phosphate-buffered saline solution, suspended in cold buffer A solution (HEPES [10 mM, pH 7.9], KCl [10 mM], EDTA [0.1 mM], EGTA [0.1 mM], DTT [1 mM], and PMSF [0.5 mM]) and allowed to swell on ice for 15 min. Cells were then subjected to lysis using a 10% solution of NP-40 (final 0.5% solution) and vigorous vortexing. Nuclei were pelleted by centrifugation at 1,500 *g* for 5 min, and the resulting supernatant (cytoplasmic fraction) was removed, and nuclei were washed twice in buffer A solution with NP-40 and were suspended in the same buffer A containing NaCl (0.5 M) to extract nuclear proteins. The extracted material was subjected to centrifugation at 15,000 *g* for 10 min, and the resulting supernatant was designated as the nuclear fraction. Nuclear protein extracts were solubilized with 1.0% sodium dodecyl sulfate buffer, and the resulting protein lysates were subsequently subjected to polyacrylamide gel electrophoresis on a 4%–15% gel gradient (Bio-Rad, Hercules, CA). Proteins were then transferred onto a polyvinylidene difluoride membrane and were probed with specific primary antibodies and horseradish peroxidase–conjugated secondary antibodies. Proteins were detected using the ECL system (GE Healthcare Life Bio-Sciences, Pittsburgh, PA).

### Transfection and siRNA

Transient transfections in cultured lymphoma cells were conducted using the Neon Transfection System (Thermo Fisher Scientific) and representative DLBCL cells. Pre-selected and validated control and OGT siRNAs (s16093, s16094, s16095) were purchased from ThermoFisher Scientific (Waltham, MA). The siRNAs were further validated in a representative DLBCL cell line MS (Supplementary Figure S2), and the best siRNA (#3) was selected for subsequent studies.

### Co-immunoprecipitation procedures

Antibodies were crosslinked to Dynabeads Protein A (Thermo Fisher Scientific) according to the manufacturer's directions. Cell lysates were pre-cleared with immunoglobulin G Dynabeads Protein A for 30 min at 4°C before incubation with antibody-linked Dynabeads overnight at 4°C. The immunoprecipitated Dynabead complexes were washed five times with immunoprecipitation buffer (Tris-HCl [pH 7.8, 10 mM], EDTA [1 mM], NaCl [150 mM], NaF [1 mM], 0.5% Nonidet P-40, 0.5% glucopyranoside, aprotinin [1 μg/mL], and phenylmethylsulfonyl fluoride [0.5 mM]). Proteins were eluted by boiling in protein-loading buffer and then were processed for Western blot analysis.

### Cell growth and viability assays

Cell viability was assessed using the CellTiter-Glo luminescent assay (Promega, Madison, WI). Cells were plated in triplicate (5–10 × 10^3^ cells/well) in 384-well plates with various concentrations of azaserine (20 μL total volume). Cell viability was assessed 72 h after treatment.

### Apoptosis and cell-cycle analysis

Apoptosis assays and cell-cycle analysis were performed according to previously published protocols [39]. For the apoptosis assay, cells were washed and stained with Annexin V-fluorescein isothiocyanate and propidium iodide in accordance with the manufacturer's recommendation (BD Pharmingen, San Diego, CA), and the apoptotic cells were quantified using a fluorescence-activated cell sorter and CellQuest software (BD Biosciences, San Jose, CA). For cell-cycle analysis, cells were fixed with cold ethanol overnight and then were treated with propidium iodide (2 μg/mL) and RNase before fluorescence-activated cell sorter analysis.

### Co-immunoprecipitation procedures

Antibodies were crosslinked to Dynabeads Protein A (Thermo Fisher Scientific) according to the manufacturer's directions. Cell lysates were pre-cleared with immunoglobulin G Dynabeads Protein A for 30 min at 4°C before incubation with antibody-linked Dynabeads overnight at 4°C. The immunoprecipitated Dynabead complexes were washed five times with immunoprecipitation buffer (Tris-HCl [pH 7.8, 10 mM], EDTA [1 mM], NaCl [150 mM], NaF [1 mM], 0.5% Nonidet P-40, 0.5% glucopyranoside, aprotinin [1 μg/mL], and phenylmethylsulfonyl fluoride [0.5 mM]). Proteins were eluted by boiling in protein-loading buffer and then were processed for Western blot analysis.

### *In vitro* ^99m^Tc-ECG cellular uptake assay

Radiosynthesis of ^99m^Tc-ECG was previously demonstrated [24, 25]. We assessed radioactivity by using a gamma counter (Packard Instruments, Meriden, CT).OCI-LY10 cells were plated in 6-well tissue culture plates (2 × 10^6^ cells/well) and were incubated with ^99m^Tc-ECG (0.05 mg/well, 8 μCi/well) or the control agent ^99m^Tc-ethylenedicysteine (0.05 mg/well, 8 μCi/well) for 0–4 h. After incubation, cells were washed twice with ice-cold phosphate-buffered saline solution. Cells were then collected, and the radioactivity of the cells was measured in triplicate. Radioactivity was expressed as percentage of cellular uptake (mean ± standard deviation).

### *In vivo* ^99m^Tc-ECG imaging in severe combined immunodeficient mice

Female severe combined immunodeficient NOD.Cg-Prkdc^scid^I12r_g_^tm1Wj1^/SzJ mice (6 weeks old) (Jackson Laboratory, Bar Harbor, ME) were inoculated subcutaneously in the thigh of the left hind leg with OCI-LY10 lymphoma cells (10^6^ cells/mouse). *In vivo* ^99m^Tc-ECG imaging was performed 14–20 days after inoculation when tumors were approximately 1 cm in diameter. The tumor-bearing mice (n = 4) were anesthetized and injected intravenously with ^99m^Tc-ECG (300 μCi/mouse), and images were acquired at 30, 120, and 240 min after administration of tracers. Scintigraphic images were obtained either from micro-positron emission tomography (PET; Inveon, Istanbul, Turkey) embedded in the gantries coordinate PET/computed tomography data acquisition or from an M-gamma camera (Siemens Medical Solutions, Inc., Malvern, PA) equipped with a low-energy parallel-hole collimator. Computer-outlined regions of interest (in counts per pixel) between tumor and muscle tissue were used to calculate tumor-to-muscle ratios.

### Statistical analysis

Overall survival (OS) for patients was calculated from the date of diagnosis to the date of last follow-up or death, and progression-free survival (PFS) was calculated from the date of diagnosis to the progression date or death. OS and PFS curves of the various groups were analyzed using GraphPad Prism 6 (GraphPad Software, La Jolla, CA) and the Kaplan-Meier method, and differences between experiments were assessed by the log-rank (Mantel-Cox) test. *P* values < 0.05 were considered statistically significant and were determined using the Student *t*-test.

## SUPPLEMENTARY MATERIALS FIGURES


