# Supplementary material for: Targeting the hexosamine biosynthetic pathway and O-linked N-acetylglucosamine cycling for therapeutic and imaging capabilities in diffuse large B-cell lymphoma
Source: Oncotarget. 2016 Oct 3;7(49):80599–611. doi: 10.18632/oncotarget.12413 (PMC5348344; doi:10.18632/oncotarget.12413)
Supplement: Supplementary file 1 [file oncotarget-07-80599-s001.pdf]

Targeting the hexosamine biosynthetic pathway and O-linked N-acetylglucosamine cycling for therapeutic and imaging capabilities in diffuse large B-cell lymphoma

SUPPLEMENTARY FIGURES

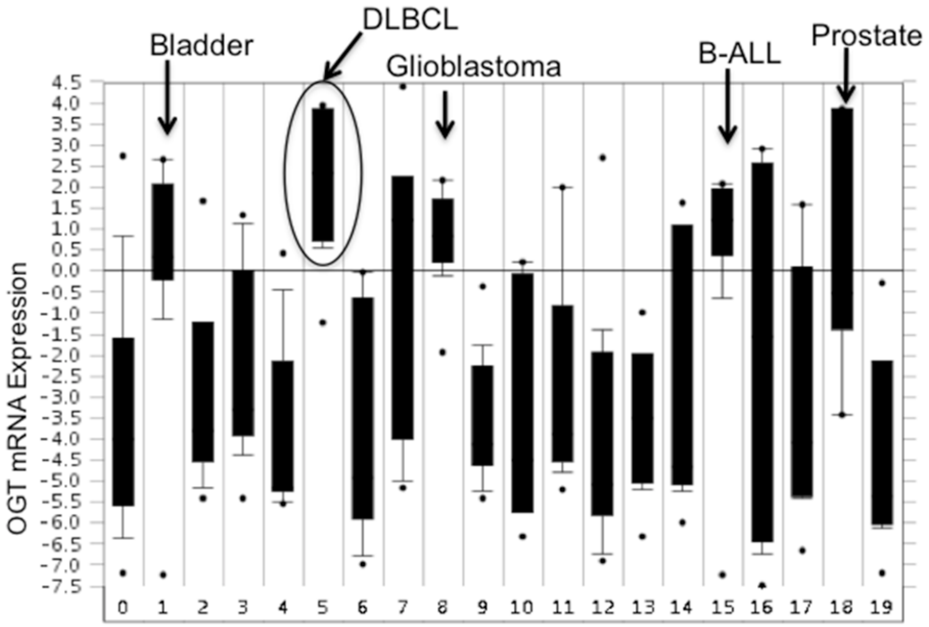

0. No value (90)
1. Acute Myeloid Leukemia (10)
2. Bladder Urothelial Carcinoma (11)
3. Breast Adenocarcinoma (12)
4. Colorectal Adenocarcinoma (12)
5. Diffuse Large B-Cell Lymphoma (11)
6. Endometrial Adenocarcinoma (10)
7. Follicular Lymphoma (11)
8. Glioblastoma (10)
9. Lung Adenocarcinoma (12)
10. Medulloblastoma (10)
11. Melanoma (10)
12. Ovarian Adenocarcinoma (12)
13. Pancreatic Adenocarcinoma (11)
14. Pleural Mesothelioma (11)
15. B-Cell Acute Lymphoblastic Leukemia (10)
16. T-Cell Acute Lymphoblastic Leukemia (10)
17. Prostate Adenocarcinoma (10)
18. Prostate Carcinoma (4)
19. Renal Cell Carcinoma (11)

Supplementary Figure S1: Overexpression of O-linked N-acetylglucosamine transferase (*OGT*) mRNA in diffuse large B-cell lymphoma (DLBCL) and other cancers. Analysis of *OGT* mRNA expression in different types of cancers using Oncomine microarray data.(40) The Student *t*-test was performed on Oncomine results. The boxes represent the 25th through 75th percentiles, the horizontal lines represent the medians, the whiskers represent the 10th and 90th percentiles, and the asterisks represent the ranges. DLBCL is one of the few cancers that have high expression of *OGT* mRNA. Abbreviation: B-ALL, B-cell acute lymphoblastic leukemia.

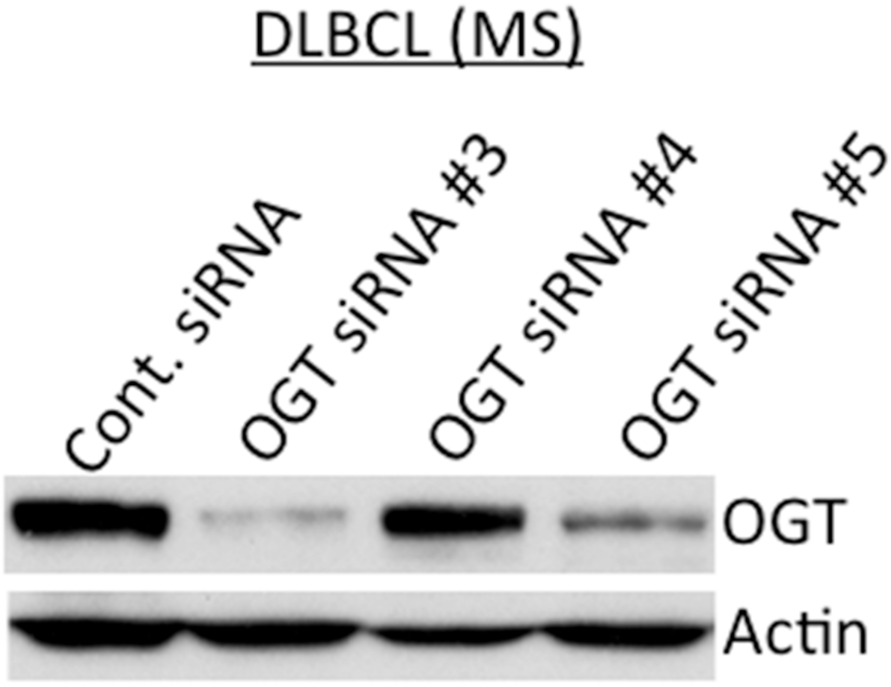

Supplementary Figure S2: Validation of OGT siRNA in a representative DLBCL cell line MS. MS cells were transfected with a control siRNA or with 3 pre-selected and validated OGT siRNAs (#3, #4, and #5) for 48 h. Whole cell extracts were purified and subjected to western blot for OGT or Actin (loading control). The best OGT siRNA (#3) was selected for further studies.

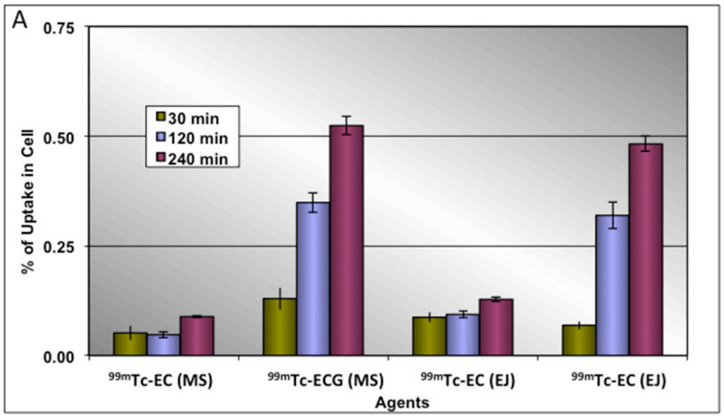

B

Mouse #	1	2	3	4	Average
Agent	^{99m}Tc-ECG (0.01 mg)	^{99m}Tc-ECG (0.01 mg)	^{99m}Tc-ECG (0.01 mg)	^{99m}Tc-ECG (0.01 mg)	
T/M	4.71	2.85	2.44	2.57	3.14

Supplementary Figure S3: *In vitro* and *in vivo* uptake of technetium-99m-ethylenedicysteine (^{99m}Tc-EC) and technetium-99m-ethylenedicysteine-N-acetylglucosamine (^{99m}Tc-ECG) in diffuse large B-cell lymphoma (DLBCL) cells. A. MS and EJ cells were plated in 6-well tissue culture plates (2 × 10^6 cells/well) and were incubated with ^{99m}Tc-ECG (0.05 mg/well, 8 µCi/well) or the control agent ^{99m}Tc-ethylenedicysteine (0.05 mg/well, 8 µCi/well) in a time dependent manner (30, 120, and 240 min). After incubation, cells were washed twice with ice-cold phosphate-buffered saline solution. Cells were then collected, and the radioactivity of the cells was measured in triplicate. Radioactivity was expressed as percentage of cellular uptake (mean ± standard deviation). **B.** The tumor-bearing mice were anesthetized and injected intravenously with ^{99m}Tc-ECG (300 µCi/mouse), and images were acquired at 30, 120, and 240 min after administration of tracers. Scintigraphic images were obtained and the computer-outlined regions of interest (in counts per pixel) between tumor and muscle tissue were used to calculate tumor-to-muscle ratios.
